# Proximal Femur Fractures in the Elderly—A Novel Modality to Predict Mortality: The Neutrophil-to-Lymphocyte Ratio

**DOI:** 10.3390/jcm12020456

**Published:** 2023-01-06

**Authors:** Omer Marom, Inbar Paz, David Segal, Guy Topaz, Nitzan Abelson, Alex Tavdi, Refael Behrbalk, Ezequiel Palmanovich, Nissim Ohana, Eyal Yaacobi

**Affiliations:** Orthopedic Department, Meir Medical Center, Kfar Saba, Affiliated to Sackler Faculty of Medicine, Tel-Aviv University, Tel Aviv 4428164, Israel

**Keywords:** comorbidity, femur, fracture, mortality, neutrophil-to-lymphocyte ratio

## Abstract

Background: The assessment and identification of elderly patients with proximal femur fractures (PFF) who are at high risk of postoperative mortality may influence the treatment decision-making process. The purpose of this study was to determine whether the neutrophil-to-lymphocyte ratio (NLR) could be used to predict postoperative mortality in the elderly population. Methods: A four-year retrospective cohort study of electronic medical records was conducted at a single tertiary care hospital between 2015 and 2018. Data from 1551 patients aged 65 years and older who underwent surgical treatment for PFF were collected and analyzed. The data included complete blood counts at admission, demographic information, underlying illnesses, type of surgery, and postoperative mortality and complications during the first year of follow-up. A survival analysis model was utilized. Results: The mean age was 90.76 ± 1.88 years, 1066 (68.7%) women. Forty-four (2.8%) patients experienced postoperative infection. A higher NLR_0_ was independently associated with higher all-cause mortality rates in patients who underwent surgical treatment for PFF (*p* = 0.041). Moreover, the mean NLR_0_ value was higher when the death occurred earlier after surgery (*p* < 0.001). Conclusions: When combined with other clinical and laboratory findings, NLR_0_ levels may serve as a potentially valuable, inexpensive, and reliable prognostic biomarker to improve risk stratification for elderly patients who are candidates for PFF surgery. Furthermore, with additional research, we could potentially develop a treatment algorithm to identify patients at high risk of postoperative mortality.

## 1. Introduction

Proximal femur fractures (PFF) are a major healthcare and financial challenge, resulting in significant morbidity, mortality, and decreased functional ability [1,2,3,4]. As the population ages, the annual number of PFF increases [3,4,5,6]. Patients with PFF have a higher risk of mortality compared to the general population at their age. Mortality rates are estimated to be 10% one month after the fracture and 20–30% a year after the fracture [7,8,9,10,11]. Currently, surgical treatment of PFF is common practice, preferably within the first 48 h of injury. Assessing mortality, morbidity, and postoperative complications in patients with PFF is a complex task as the affected population is predominantly elderly and frail, with multiple comorbidities and a low level of preoperative physical activity [12]. Due to the severe pain and disability associated with an untreated PFF, a low threshold for surgical treatment may be required. Early risk stratification helps identify PFF patients who may benefit from a non-surgical approach or a less invasive type of surgery combined with pain management and immobilization. The use of clinical scoring systems, such as the American Society of Anesthesiologists’ (ASA) physical status classification system and red blood cell distribution width (RDW) as prognostic factors, has shown efficacy in distinguishing between high-risk and low-risk patients [13,14]. To identify high-risk PFF patients, clinical, radiologic, and laboratory evaluations should be performed.

PFF and the subsequent surgical treatment may trigger an inflammatory response and induce changes in white blood cell levels, resulting in an increased neutrophil count and decreased lymphocyte count [15,16]. The neutrophil-to-lymphocyte ratio (NLR) is calculated by dividing the absolute number of neutrophils circulating in the blood by the absolute number of lymphocytes circulating in the blood, making NLR routinely available from complete blood counts. Over the past decade, NLR has been extensively studied as a rapid, widely available, and inexpensive biomarker of the systemic inflammatory response. Elevated NLR levels were found to be a significant predictor of adverse outcomes and mortality in patients with a wide range of orthopedic conditions [17,18] as well as non-orthopedic conditions such as cardiovascular [19,20,21,22,23,24], renal [25,26], COPD [27], inflammatory [28,29], oncologic [30,31,32,33], and various postoperative conditions [34]. Thus, it is reasonable to assume that NLR is a marker of frailty or poor health and that it could be used for better risk stratification in the fields of orthopedic surgery as well as internal medicine.

In our effort to further investigate the role of NLR as a prognostic factor in patients with PFF, we hypothesized that NLR at hospital admission (NLR_0_) can serve as a reliable predictor of postoperative adverse outcomes in patients with PFF undergoing surgical treatment. We specifically investigated the relationship between an elevated level of NLR_0_ and mortality and infection rates at 1, 3, 6, and 12 months postoperatively.

## 2. Materials and Methods

### 2.1. Study Setting and Population

The study was conducted at a university-affiliated tertiary care hospital. The Orthopedic Trauma Unit performs approximately 500 PFF surgeries annually. From 1 January 2015 through 31 December 2018, we collected and analyzed data from all patients aged 65 years and older who underwent bipolar cementless hemiarthroplasty, total hip arthroplasty, and closed reduction internal fixation for intracapsular and extracapsular PFFs (AO/OTA 31). An orthopedic surgery specialist used a physical examination and radiographic studies to diagnose PFF. The type of surgery was chosen according to the type of fracture and the patient’s medical condition. Patients with the following characteristics were excluded from the analysis: pathological fractures, those under 65 years of age at admission, and those who had been treated with chemotherapy, radiotherapy, or steroids within six months of admission.

### 2.2. Laboratory Measurements and Data Collection

We collected data from electronic medical records, including patients’ demographic information, underlying illnesses at the time of admission, type of surgery, and postoperative follow-up and complications. Mortality rates were obtained from the Israel Central Bureau of Statistics. Data collection was approved by the Institutional Review Board.

On admission, venous peripheral blood samples were routinely collected at the Emergency Department (ED) for complete blood count, including neutrophil and lymphocyte counts. Blood samples were delivered to a central laboratory within one hour of venipuncture and analyzed using standardized, automated kits (Adiva 2120, Siemens, Erlangen, Germany). During the one-year follow-up period, blood counts were retaken at routine clinic visits to the family physician. Since NLR was not a routinely derived marker, we calculated it for the purpose of this study.

### 2.3. Clinical Endpoints and Terms

Clinical endpoints included all-cause mortality and postoperative infection within the first year of surgery. Specialized orthopedic surgeons diagnosed the latter, taking into account blood test results, purulent wound discharges, positive wound cultures, abnormal swelling of the surgical site, local heat, erythema, and pain. Patients were divided into two groups. The first group included patients who did not die during the first postoperative year. The second group included patients who died during the first postoperative year. The second group was further divided into sub-groups based on when the patients passed away: within the first month, three months, six months, or twelve months of surgery. The mean NLR_0_ of Group 1 was compared to that of each of the sub-groups.

### 2.4. Statistical Analysis

We used descriptive statistics to present raw data. We used the student *t* test to compare continuous variables, and the Chi^2^ test to compare categorical variables. We used the Pearson’s correlation coefficient to evaluate correlations between two continuous variables. A Cox regression multivariable survival model was then constructed: the first year all-cause mortality was set as the dependent variable. Variables which have been found to have a statistically significant association with the dependent variable were introduced as the covariables. The hazard ratios for each variable were reported, along with the 95% confidence intervals (CI) and the *p* values. We performed a Receiver Operating Characteristic (ROC) curve analysis to detect the best cut-off to predict the first-year mortality (as the categorical variable) with the NLR_0_ (as the continuous variable). Then we used this cut-off to categorize the patient population into two groups according to their NLR_0._ Following this categorization we constructed and analyzed Kaplan-Meier plot where the two groups were compared for survival. We regarded a beta of 0.05 and an alpha of 0.8 as statistically significant. The data was collected and analyzed with an SPSS V28.0 software (Armonk, NY, USA).

## 3. Results

### 3.1. Inclusion and Exclusion

**Figure 1:**
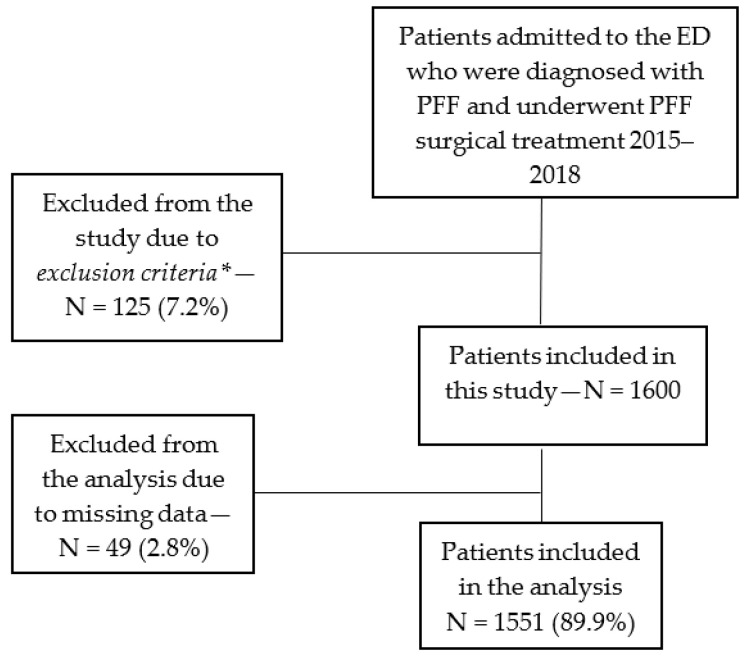
Study population flowchart (inclusion and exclusion). * Exclusion criteria: patients diagnosed with pathological fracture, younger than age 65 years at admission or after recent chemotherapy, radiotherapy, or steroid therapy.

During the study period, 1725 patients underwent surgical treatment for PFF at our medical center. Following an exclusion process a total of 1551 (89.9%) patients with PFF were included in the analysis (Figure 1).

### 3.2. Patient Baseline Characteristics

The mean baseline NLR_0_ of the cohort was 8.51 ± 6.52. The mean age was 90.76 ± 1.88 years and was not found to have a statistically significant correlation with the NLR_0_ level (r = 0.005, *p* = 0.83). Female patients made up the majority of the cohort (915, 69.3%), and their mean NLR_0_ level was lower than that of male patients (7.92 ± 5.52 vs. 9.83 ± 8.17 respectively, *p* < 0.001, Table 1). The mean NLR_0_ was also found to be statistically significantly lower in patients with CHF (*p* = 0.014) and COPD (*p* = 0.023, Table 1). 

### 3.3. Surgical Procedures

There was little evidence of association between surgical procedures and the one- year mortality rate (*p* = 0.058), nor with the one-, three-, and six-month mortality rates (*p* = 0.314, *p* = 0.148, and *p* = 0.071, respectively), although patients who underwent a THA had a comparably lower mortality rate (Table 2). There was no significant difference in NLR_0_ between patients who have been operated by residents or senior surgeons (*p* = 0.144). In addition, there was no significant correlation between NLR_0_ and the duration of surgery (r = 0.045, *p* = 0.079) or the duration of hospital stay (r = −0.021, *p* = 0.414). The associations between the NLR_0_ and various comorbidities is presented in Table 1.

### 3.4. NLR_0_ Postoperative Mortality and Infection

**Figure 2:**
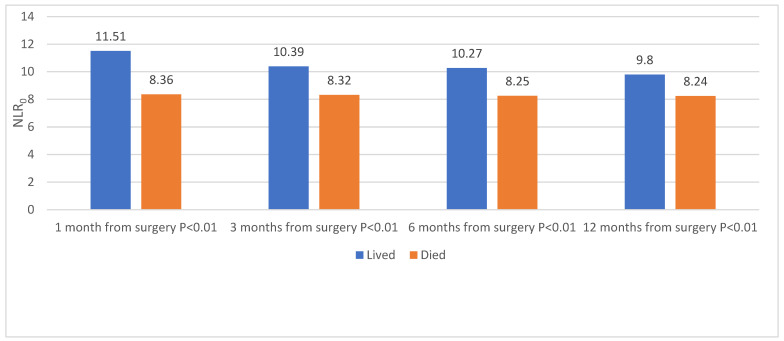
Primary outcome (all-cause mortality) as a function of NLR. All-cause mortality during the first year following a proximal femur fracture surgery in 1551 elderly patients (altogether: 1278 (82.4%) lived, 273 (17.6%) died). NLR_0_: neutrophile/Lymphocyte ratio.

The overall mortality rate one year after surgery was 17.6% (273 patients). Patients who died during the first post-operative year had a higher mean NLR_0_ in all of the study’s four timepoints (1, 3, 6, and 12 months, *p* < 0.001, Figure 2). The mean NLR_0_ value was higher when the death occurred early after surgery (*p* = 0.009, Figure 2). Forty-three (2.8%) patients suffered from postoperative infection, but this complication had no association with the first-year mortality rate (*p* = 0.495, Table 2).

The multivariable Cox regression analysis included variables that have been found to be associated with a higher NLR_0_ in the preliminary univariable analysis (Table 2): sex, CRF, IHD, AF/PAF, colon cancer, smoking history, WBC, Hemoglobin, platelet count, MCV, RDW, Urea, Creatinine, and Albumin. The HR (hazard ratio) for NLR_0_ was found to be 1.024 (95% confidence interval 1.001 to 1.047, *p* = 0.041 (Table 3). Additional factors (Table 3) that have been found to have a statistically significant HR were sex (female being protective, *p* = 0.014), higher RDW (*p* = 0.026), high urea (*p* < 0.001). and low albumin (*p* < 0.001).

**Figure 3:**
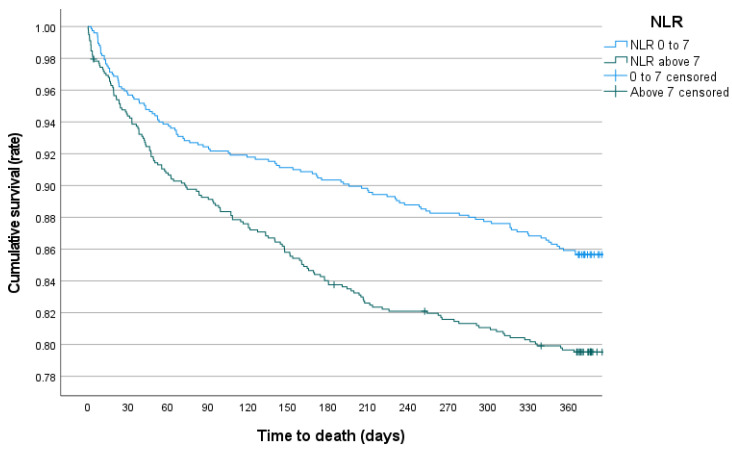
Kaplan-Meier survival function: All-cause mortality following proximal femur fracture fixation surgery in an elderly population (*p* < 0.001).

A ROC curve revealed that a cut-off of seven would provide the optimal distinction between patients with comparatively low or high NLR_0_ when plotted against the first-year mortality (*p* < 0.001, AUC = 0.564). A Kaplan-Meier survival curve revealed a statistically significant difference between the survival distributions of the two groups (*p* < 0.001 for the Log Rank test of equality, Figure 3). 

### 3.5. Limitations

This study has several limitations. First, due to the lack of laboratory data, NLR was only calculated on admission, with no assessment of possible changes during the postoperative period at the hospital. Second, due to the retrospective nature of this study, the Cox regression analysis was unable to account for all the differences between those who survived and those who died during the first post-operative year. Third, this study referred only to the patients treated surgically, with no comparison between patients treated operatively vs. non-operatively. Further studies comparing these two groups will help to understand the role of NLR_0_ in the treatment decision-making process. Additional factors that have been found in association with the first post-operative year mortality were male sex, high urea, and low albumin levels. These findings are of importance but investigating them was beyond the scope of this study. Further studies in this regard should be performed to better understand their role as prognostic factors in the studied population. Finally, the cut-off for elevated NLR in the majority of the studies mentioned above was 5.5, and 18 for severely elevated NLR. In our study, the mean NLR for all patients was relatively high, at 8.2, and the optimal cutoff was set at 7. This might be explained by the characteristics of our study population, which consisted of older female adults who have been found in this study to have a higher NLR_0_ values than men.

## 4. Discussion

The main finding of this study is that higher NLR_0_ was independently associated with a higher rate of all-cause mortality in patients who underwent surgery for PFF at one month, three months, six months, and twelve months postoperatively. Furthermore, this study demonstrated that the mean level of NLR_0_ was higher when the death occurred earlier after surgery. A higher NLR_0_ did not correlate with a higher rate of postoperative infection. 

The data on the association between NLR_0_ and the systemic inflammatory response is spared compared to that on CRP, IL-6, or other similar markers. The inflammatory response to trauma is complex, and studies have demonstrated that a hypo-inflammatory response, particularly in the late stages of trauma, may contribute to poor outcomes, including infection. In studies with a small sample of PFF patients, NLR_0_ has been identified as a marker for poor short- and medium-term postoperative outcomes. Alexandru L et al. investigated the association between NLR_0_ and diaphyseal fractures of the humerus, femur, and tibia in 148 patients. They discovered that patients with femur fractures had significantly higher NLR_0_ and that NLR_0_ was associated with the duration of hospitalization [35]. Fisher A et al., investigated the relationship between NLR_0_ and the presence of fractures and comorbidities, as well as the prognostic value of NLR_0_ for short-term outcomes in 415 orthogeriatric patients. They identified NLR_0_ > 5 as an independent predictor of postoperative myocardial injury and NLR_0_ > 8.5 as a predictor of infection and in-hospital mortality [36]. In another study by Temiz A et al., an increased NLR_0_ was associated with higher one-year mortality in 50 female patients with PFF [37]. In a study by Ozbek et al. on patients who underwent proximal femur fracture fixation by intramedullary nails, NLR_0_ > 5.25 was significantly associated with a higher rate of mortality one-year postoperatively [38]. 

NLR plays a highly significant role in different postoperative manifestations in orthopedic and non-orthopedic surgeries that were not demonstrated in this specific study as presented in recent studies by Melinte R. et al. and Pasqui E. et al., respectively [39,40]. 

Complementary to this current study in which the presentation of NLR (Day 0) was investigated, several studies examined the importance of the NLR levels that were measured during the post-operative period as a prognostic factor in patients with PFF. In a study of 247 patients with PFF, Forget P et al. determined that the fifth postoperative day NLR_5_ could be a risk factor for postoperative mortality and cardiovascular complications [41]. In a study of 286 patients with PFF by Forget P et al., a score composed of age, gender, NLR_5_, and CRP protein on the fifth postoperative day (CRP_5_) was predictive of mortality one-year after surgery [42]. In a study of 132 patients with PFF, Atlas A et al. demonstrated that the rate of increase in NLR values in the postoperative period is a predictor of morbidity and mortality [43]. Wasko MK et al. measured NLR and CRP levels pre-and post-total knee or hip arthroplasty in 387 patients. NLR levels had returned to preoperative values by the fifth postoperative day, and NLR had a more rapid postoperative kinetic pattern when compared to CRP [44]. While the investigation of the post-operative NLR levels was beyond the scope of the current study, these articles further demonstrate the role of the NLR values as indicators of a possible inferior outcome in PFF surgery.

Previous studies describing the link between NLR and adverse clinical outcomes in patients with PFF used small sample sizes and focused primarily on short-term outcomes. Our study included a larger sample of patients with PFF (N = 1527) and assessed both short-and medium-term outcomes. In contrast to the current study which did not demonstrate a significant association between NLR and postoperative infection, Xu H. et al. showed that NLR levels have been associated with infection but were not as reliable as the CRP levels [45]. Altogether, the current study supports the existing cumulative body of literature stating that higher NLR_0_ values have been indicative of inferior surgical outcomes.

Several mechanisms could explain the association between elevated NLR_0_ and high mortality rates following PFF surgery. Increased NLR reflects a systemic inflammatory response in which the neutrophil count is high while the lymphocyte count is low. Ozturk et al. assessed the relationship between NLR_0_ levels and bone mineral density in a cross-sectional study of 1635 elderly individuals and identified NLR_0_ as an independent predictor of osteoporosis [46]. Recent evidence suggests that inflammation may play a critical role in bone remodeling and the pathogenesis of osteoporosis [47,48] and atherosclerosis [49], which may partially explain the relationship between NLR_0_ as a marker of systemic inflammation and increased postoperative mortality following PFF surgery.

## 5. Conclusions

Our study demonstrated that elderly patients undergoing surgery for PFF who have an elevated NLR level at the time of admission could be at higher risk of both short-and medium-term postoperative mortality. A specific scale of NLR values has yet to be developed. However, we believe that these preliminary results warrant a larger database study to evaluate this biomarker and potentially contribute to the development of an algorithm or tool to help distinguish between patients who may benefit from surgical treatment and those who will not. Along with other clinical and laboratory findings, NLR_0_ could be a valuable, simple, and inexpensive prognostic biomarker that aids in mortality calculations, risk stratification, and treatment selection for patients with PFF. 

## Figures and Tables

**Table 1:** Neutrophile/lymphocyte ratio (NLR_0_) upon presentation of 1551 elderly patients with a proximal femur fracture. Values are presented as a mean ± standard deviation. CHF, chronic heart failure; CRF, chronic renal failure; DM, diabetes mellitus; COPD, chronic obstructive pulmonary disease; HTN, hypertension; IHD, ischemic heart disease; AF, atrial fibrillation; PAF, paroxysmal atrial fibrillation.

Variable	Present	Absent	*p* Value
Sex male (n = 473, 31%)	7.92 ± 5.52	9.83 ± 8.18	<0.001
CHF (n = 393, 25.7%)	7.81 ± 5.17	8.75 ± 6.91	0.014
CRF (n = 106, 6.9%)	8.58 ± 6.47	8.5 ± 6.52	0.91
DM (n = 310, 20.3%)	7.8 ± 5.62	8.67 ± 6.72	0.52
COPD (n = 66, 4.3%)	10.29 ± 8.41	8.43 ± 6.41	0.023
HTN (n = 529, 34.6%)	8.42 ± 6.02	8.56 ± 6.77	0.692
Dyslipidemia (n = 254, 16.6%)	8.57 ± 6.49	8.5 ± 6.53	0.867
IHD (n = 164, 10.7%)	6.67 ± 6.99	8.49 ± 6.46	0.73
AF/PAF (n = 117, 7.7%)	8.56 ± 6.65	8.50 ± 6.51	0.936
Breast cancer (n = 32, 2.1%)	7.79 ± 5.56	8.52 ± 6.54	0.532
Colon cancer (n = 12, 0.8%)	8.39 ± 7.45	8.51 ± 6.51	0.951
Smoking (n = 118, 7.7%)	8.57 ± 6.51	8.5 ± 6.52	0.919

**Table 2:** Patients who underwent surgery for a proximal femur fracture. Categorical variables are presented as absolute counts with rates, and continuous variables are presented as mean ± standard deviation. CHF, chronic heart failure; CRF, chronic renal failure; DM, diabetes mellitus; COPD, chronic obstructive pulmonary disease; HTN, hypertension; IHD, ischemic heart disease; AF, atrial fibrillation; PAF, paroxysmal atrial fibrillation; CRIF, closed reduction internal fixation; THA, total hip arthroplasty; HA, hemi arthroplasty; MCV, mean corpuscle volume; RDW, red blood cell distribution; MPV, mean platelet volume.

Variable	Lived Until at Least One Year after Surgery (*n* = 1278, 82.4%)	Died during the First Year after Surgery (*n* = 273, 17.6%)	Total (1551, 100%)	*p* Value
Demographics				
Age	90.76 ± 1.91	90.76 ± 1.75	90.76 ± 1.88	0.783
Survival among sex group:				<0.001
Male	363 (74.8%)	122 (25.2%)	485 (100%, 31.3% of total)	
Female	915 (85.8%)	151 (14.2%)	1066 (100%, 68.7% of total)	
Survival among patients with comorbidities				
CHF	320 (79.8%)	81 (20.2%)	401 (100%, 25.9% of total)	0.067
CRF	80 (72.1%)	31 (27.9%)	111 (100%, 7.2% of total)	0.003
DM	259 (81.2%)	60 (18.8%)	319 (100%, 20.6% of total)	0.288
COPD	55 (82.1%)	12 (17.9%)	67 (100%, 4.3% of total)	0.525
HTN	438 (81.3%)	101 (18.7%)	539 (100%, 34.8% of total)	0.215
Dyslipidemia	202 (78%)	57 (22%)	259 (100%, 16.7% of total)	0.27
IHD	126 (74.1%)	44 (25.9%)	170 (100%, 11% of total)	0.003
AF/PAF	88 (72.7%)	33 (27.3%)	121 (100%, 7.8% of total)	0.004
Breast cancer	28 (84.8%)	5 (15.2%)	33 (100%, 2.1% of total)	0.463
Colon cancer	7 (58.3%)	5 (41.7%)	12 (100%, 0.8% of total)	0.044
Smoking	109 (89.3%)	13 (10.7%)	122 (100%, 7.9% of total)	0.02
Survival by surgery features				
Type of operation				0.058
CRIF	876 (81.9%)	194 (18.1%)	1070 (100%, 69.2% of total)	
THA	66 (93%)	5 (7%)	71 (100%, 4.6% of total)	
HA	334 (82.3%)	72 (17.7%)	406 (100%, 26.2% of total)	
Duration of surgery (min)	74.84 ± 62.43	73.99 ± 46.05	74.69 ± 59.85	0.831
Surgeon-Attending orthopedic surgeon	773 (83.5%)	153 (16.5%)	926 (100%, 59.7% of total)	0.096
Postoperative infection	35 (81.4%)	8 (18.6%)	43 (100%, 2.8% of total)	0.495
Second PF surgery	46 (76.7%)	14 (23.3%)	60 (100%, 3.9% of total)	0.155
Blood laboratory tests				
NLR_0_	8.24 ± 6.05	9.8 ± 8.28	8.51 ± 6.52	<0.001
White blood cells	10.46 ± 4.35	1.058 ± 4.49	10.48 ± 4.51	0.68
Hemoglobin	11.11 ± 1.75	10.59 ± 1.68	11.02 ± 1.75	<0.01
Platelets	214.31 ± 73.32	221.6 ± 98.58	215.59 ± 78.67	0.163
MCV	89.49 ± 6.11	91.05 ± 7.4	89.77 ± 6.39	<0.01
RDW	14.24 ± 1.5	14.95 ± 1.99	14.37 ± 1.62	<0.01
MPV	8.32 ± 1.2	8.25 ± 1.11	8.31 ± 1.19	0.402
Lymphocytes	1.41 ± 2.41	1.15 ± 0.69	1.37 ± 2.21	0.087
Urea	48.42 ± 22.28	67.63 ± 38.6	51.76 ± 26.86	<0.001
Creatinine	1.07 ± 0.59	1.46 ± 1.16	1.14 ± 0.74	<0.001
Calcium	8.53 ± 0.58	8.47 ± 0.86	8.52 ± 0.64	0.282
Albumin	3.19 ± 0.65	3.01 ± 0.38	3.16 ± 0.36	<0.001

**Table 3:** A Cox regression analysis: The dependent variable is a death (yes or no) during the first year following a surgery for a proximal femur fracture in the elderly population. The covariables have been detected as having a statistically significant association with the dependent variable on a preliminary univariate analysis. CRF, chronic renal failure; IHD, ischemic heart disease; AF, atrial fibrillation; PAF, paroxysmal atrial fibrillation; MCV, mean corpuscle volume; RDW, red blood cell distribution.

Variable	Hazard Ratio	95% Confidence Interval		*p* Value
		Lower Limit	Upper Limit	
Demographics and comorbidities				
Sex	0.632	0.438	0.912	0.014
CRF	0.734	0.414	1.3	0.289
IHD	1.459	0.924	2.306	0.105
AF/PAF	1.175	0.681	2.028	0.561
Colon cancer	1.428	0.464	4.398	0.534
Smoking	0.556	0.241	1.283	0.169
Blood laboratory tests				
NLR_0_	1.024	1.001	1.047	0.041
Hemoglobin	0.996	0.895	1.108	0.938
MCV	1.026	0.999	1.053	0.063
RDW	1.125	1.014	1.249	0.026
Urea	1.021	1.012	1.03	<0.001
Creatinine	0.842	0.65	1.089	0.19
Albumin	0.34	0.218	0.53	<0.001

## Data Availability

The data presented in this study are available on request from the corresponding author. The data are not publicly available due to privacy issues.
